# Synthesis of PNVP-Based Copolymers with Tunable Thermosensitivity by Sequential Reversible Addition–Fragmentation Chain Transfer Copolymerization and Ring-Opening Polymerization

**DOI:** 10.3390/polym9060231

**Published:** 2017-06-18

**Authors:** Yi-Shen Huang, Jem-Kun Chen, Tao Chen, Chih-Feng Huang

**Affiliations:** 1 Department of Chemical Engineering, National Chung Hsing University, 250 Kuo Kuang Road, Taichung 40227, Taiwan; yishen617@gmail.com; 2 Department of Materials Science and Engineering, National Taiwan University of Science and Technology, 43, Sec. 4, Keelung Road, Taipei 10607, Taiwan; jkchen@mail.ntust.edu.tw; 3 Department of Polymer and Composite, Ningbo Institute of Materials Technology and Engineering Chinese Academy of Sciences, Zhongguan West Road 1219, Ningbo 315201, China; tao.chen@nimte.ac.cn

**Keywords:** poly(*N*-vinylpyrrolidone), RAFT copolymerization, ring-opening polymerization, thermosensitive block copolymer, poly(ε-caprolactone)

## Abstract

Through the reversible addition–fragmentation chain transfer (RAFT) copolymerization of 3-ethyl-1-vinyl-2-pyrrolidone (C_2_NVP) and *N*-vinylpyrrolidone (NVP), a series of well-defined P(C_2_NVP-*co*-NVP) copolymers were synthesized (*M*_n_ = ca. 8000 to 16,000 and *M*_w_*/M*_n_ <1.5) by using a difunctional chain transfer agent, *S*-(1-methyl-4-hydroxyethyl acetate) *O*-ethyl xanthate (MHEX). Copolymerizing kinetics and different monomer ratio in feeds were conducted to study the apparent monomer reaction rate and reactivity ratios of NVP and C_2_NVP, which indicated similar reaction rates and predominantly ideal random copolymers for the two monomers. The *T*_g_s of the obtaining P(C_2_NVP-*co*-NVP) copolymers significantly corresponded to not only molecular weights MWs but also copolymer compositions. These copolymers presented characteristic lower critical solution temperatures (LCST) behavior. We then studied the cloud points (CPs) of the copolymers with varying MWs and compositions. With different MWs, the CPs were linearly decreased from ca. 51 to 45 °C. With different compositions, the CPs of the copolymers decreased from ca. 48 to 29 °C with C_2_NVP content (i.e., from 60.8 to 89.9 mol %). Fitting the CPs by the theoretical equation, the result illustrated that the introduction of more hydrophobic units of C_2_NVP suppressed the hydrophilic interaction between the polymer chain and water. We then successfully proceeded the chain extension through the ring-opening polymerization (ROP) of ε-caprolactone (CL) to the synthesis of a novel P(C_2_NVP-*co*-NVP)-*b*-PCL amphiphilic block copolymer (*M*_n,NMR_ = 14,730 and *M*_w_/*M*_n_ = 1.59). The critical micelle concentration (CMC) of the block copolymer had a value of ca. 1.46 × 10^−4^ g/L. The block copolymer micelle was traced by dynamic light scattering (DLS), obtaining thermosensitive behaviors with a particle size of ca. 240 nm at 25 °C and ca. 140 nm at 55 °C, respectively.

## 1. Introduction

Thermoresponsive polymers that exhibit lower critical solution temperatures (LCST) in aqueous solution are among the most important water-soluble polymers. These polymers have found versatile applications in various fields, such as catalysis, intelligent drug delivery, and tissue engineering [1,2,3,4,5]. A key feature of these polymers is a reversible phase transition in an aqueous solution, resulting from a reversible change of polymer conformation at a certain temperature (i.e., cloud point (CP)). In general, this phenomenon is based on the diminishing entropy with increasing temperature caused by the immobilization of the solvent molecules on the surface of the dispersed polymer chain. Based on the side group structures, these polymers can be mainly categorized into four classes: amide [6,7,8], ether [9,10], oxazoline [11,12], and amino acrylate [13,14]. For the rational design of a new type of thermoresponsive polymer, a key is to reveal its significant and tunable phase transition property. Poly(*N*-isopropyl acrylamide) (PNIPAM) is the most widely studied amide-type thermoresponsive polymer, which possesses a secondary amide group with a hydrophobic isopropyl group in the side chain. In an aqueous solution, PNIPAM performs a sharp and reversible phase transition at a temperature that is close to our body temperature. The CPs of homopolymers containing an amide side group, such as poly(*N*-vinylcaprolactam) (PNVCL) and poly(*N*,*N*-diethylacrylamide) (PDEA), generally display significant and simple thermosensitive behaviors. The Cai group synthesized a series of pyrrolidone-based (co)polymers, such as poly[*N*-(2-methacryloyloxyethyl)pyrrolidone] (PNMEP), poly[*N*-(3-acryloyloxypropyl)pyrrolidone] (PNAPP), and poly[*N*-(3-methacryloyloxypropyl)-pyrrolidone] (PNMPP) [15,16]. They interestingly demonstrated that the fully hydrated state of the polymers in D_2_O had a phase transition occur at the cloud point, and that further heating leads to dehydration and separation from the D_2_O. The Ritter and Li groups found that ethyl-substituted *N*-vinylpyrrolidone (NVP) at the 3-position (i.e., C_2_NVP) featured a thermoresponsive polymer (i.e., PC_2_NVP) with a significant CP in water. The CP of PC_2_NVP is around 25 °C [8,17,18,19], which can be adjusted by free radical copolymerization with NVP [19]. The exploration of the P(C_2_NVP-*co*-NVP) copolymers’ properties has not been addressed. Thus, tuning thermosensitive behaviors is also an important and practical issue. Generally, control over the LCST of thermoresponsive polymers has been achieved by manipulating either the chemical factors, such as polymer structure, composition, chain-end groups, and polymer molecular weights, or the physical factors, such as additives and organic solvents [20,21,22,23]. Furthermore, the relationship between CP and composition was discussed by the theoretical Kwei equation [24].

In the past few decades, significant efforts have been devoted to the development of living polymerizations, including reversible-deactivation radical polymerizations (RDRPs) [25], living polycondensations [26,27,28,29], ring-opening polymerizations (ROPs) [30], anionic [31,32]/cationic [33] polymerizations, and ring-opening metathesis polymerizations (ROMPs) [34]. These methods provide mighty tools for the preparation of unprecedented nanomaterials [35,36]. Through free radical chemistry, RDRPs render innovative polymeric materials from a variety of monomers, leading to excellent control over chain functionality, molecular weight (MW), and molecular weight distribution (MWD). The most widely studied RDRP systems include nitroxide-mediated radical polymerization (NMRP) [37], reversible addition–fragmentation transfer (RAFT) polymerization [38], and atom transfer radical polymerization (ATRP) [39,40]. In addition, some other RDRP systems have been also reported [41,42,43,44]. For the so-called “more reactive monomers” (MAMs), the propagating radicals can be stabilized via the resonance or inductive effect. The MAMs can be generally proceeded to control polymerization through an ATRP mechanism [45,46,47]. Although there has been significant progress and good design in the ATRP catalytic systems [48,49,50], challenges still remain for controlling the polymerizations of “less reactive monomers” (LAMs) such as NVP and NVCL. Thus, combinations of two living polymerization mechanisms (herein: RAFT polymerization and ROP) by a dual functional initiator could provide a facile route to the synthesis of new well-defined thermosensitive (co)polymers.

**Scheme 1:**
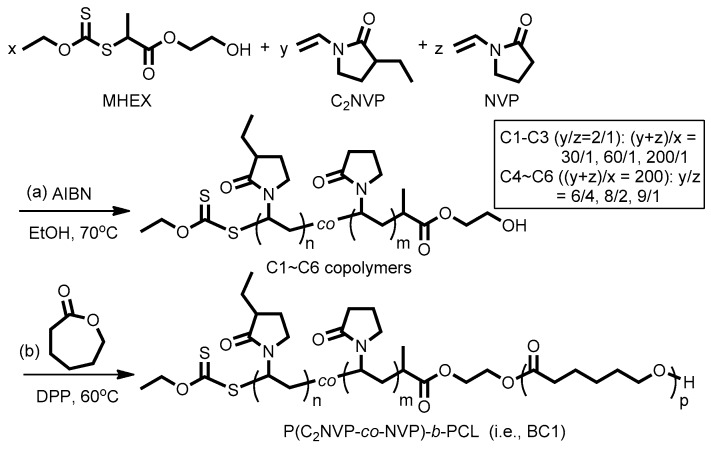
(**a**) reversible addition–fragmentation transfer (RAFT) copolymerization of *N*-vinylpyrrolidone (NVP) and 3-ethyl-1-vinyl-2-pyrrolidone (C_2_NVP) and (**b**) ring-opening polymerization (ROP) of ε-caprolactone (CL).

As shown in Scheme 1, we first synthesize a series of P(C_2_NVP-*co*-NVP) copolymers through RAFT polymerization with the dual functional MHEX chain transfer agent. Our attempt is to obtain well-defined PNVP-based copolymers (i.e., C1–C6 copolymers) with tunable thermosensitivity and chain-end functionality. Detailed studies of kinetic characterization during RAFT copolymerization, the thermal properties of the copolymers in solid-sate, and the cloud point (CP) phenomena of the copolymers in water were discussed. We further proceeded the chain extension of P(C_2_NVP-*co*-NVP)-OH through the ROP of ε-caprolactone (CL) to the synthesis of a novel P(C_2_NVP-*co*-NVP)-*b*-PCL amphiphilic block copolymer. Eventually, block copolymer micelle was prepared and traced by dynamic light scattering (DLS) to demonstrate the thermosensitive behaviors of the novel P(C_2_NVP-*co*-NVP)-*b*-PCL amphiphilic block copolymer.

## 2. Materials and Methods

### 2.1. Materials

Ethylene glycol (EG, 99%), pyridine (99%), 2-bromopropanoyl bromide (98%), potassium ethyl xanthate (97%), 1-bromoethane (98%), azobisisobutyronitrile (AIBN, 99%), 2.0 M lithium diisopropylamide (LDA) in tetrahydrofuran (THF), diphenyl phosphate (DPP, 97%), and anhydrous magnesium sulfate (MgSO_4_, 99%) were purchased from Sigma-Aldrich (St. Louis, MO, USA) and were used without purification. The solvents *N*-vinylpyrrolidone (NVP, 99%) and ε-caprolactone (CL, 99%) were distilled and stored in a molecular sieve prior to use.

### 2.2. Synthesis of 2-Hydroxyethyl 2-Bromopropanoate (HEB)

As shown in Scheme S1a: 2-Bromopropanoyl bromide (10.2 g, 47.4 mmol) was dissolved in 50 mL of anhydrous THF and added dropwise to a solution of ethylene glycol (150 g, 2.42 mol), pyridine (3.75 g, 47.4 mmol), and 500 mL anhydrous THF at 0 °C within 1 h. After the reaction finished, the mixture was diluted by hydrochloric acid to obtain a solution with a pH equal to 2. The aqueous layer was then extracted three times with dichloromethane (DCM). The organic phase was combined and further washed with DI water. The organic layer was dried over anhydrous MgSO_4_ and evaporated under vacuum. A light pale yellow oil product (i.e., HEB) was obtained (yield = 5.2 g (55.8%); ^1^H NMR (400 MHz, CDCl_3_, δ = ppm): 8.02–7.09 (d, *J* = 9 Hz, 2H), 7.40–7.36 (d, *J* = 9 Hz, 2H), 4.78 (s, 2H), 3.89 (s, 3H), 0.94 (s, 9H), and 0.10 (s, 6H)).

### 2.3. Synthesis of S-(1-Methyl-4-hydroxyethyl Acetate) O-Ethyl Xanthate (MHEX)

As consecutively shown in Scheme S1a: HEB (4.96 g, 25.3 mmol) was dissolved in 50 mL acetone and added dropwise to a solution of potassium ethyl xanthate (4.51 g, 28.3 mmol) with 50 mL acetone at room temperature in 30 min. After the reaction finished, the heterogeneous mixture was filtrated to remove the solid salt, and washed by acetone. The filtrate was then condensed by vacuum and diluted by DCM. The organic phase was washed with DI water, dried over MgSO_4_, and evaporated under vacuum. A pale yellow oil product (i.e., MHEX) was obtained (yield = 5.23 g (87.1%); ^1^H NMR (400 MHz, CDCl_3_, δ = ppm (see Figure S1a in the Supporting Information)): 1.42 (t, 3H), 1.60 (d, 3H), 1.85–2.00 (m, 1H), 3.80–3.90 (m, 2H), 4.28 (qt, 2H), 4.42 (q, 1H), and 4.64 (q, 2H)).

### 2.4. Synthesis of 3-Ethyl-1-vinyl-2-pyrrolidone (C_2_NVP)

The synthetic procedures were modified according to the previous study [17,18]. As shown in Scheme S1b: NVP (10 g, 90 mmol) was dissolved in 50 mL of dry THF, and added dropwise to 45 mL of a 2.0 M LDA/THF solution (90 mmol) at 0 °C. After the addition, 1-bromoethane (10.3 g, 95 mmol) with 50 mL of dry THF was then added dropwise. After the reaction finished, the mixture was quenched by DI water (100 mL). DCM was added to extract the aqueous for three times. The organic phase was collected, dried over MgSO_4_, and evaporated under vacuum. The crude product was purified by flash chromatography (hexane/ethyl acetate = 4:1 (*v*/*v*)), and yielded 4.48 g (35.8%) of C_2_NVP as a pale yellow oil (^1^H NMR (400 MHz, CDCl_3_, δ = ppm (see Figure S1b in the Supporting Information)): 0.98 (t, *J* = 7.5 Hz, 3H), 1.45 (m, 1H), 1.69–1.96 (m, 2H), 2.22–2.54 (m, 2H), 3.33–3.56 (m, 2H), 4.38 (d, J = 16.0 Hz, 1H), 4.43 (d, *J* = 9.2 Hz, 1H), 7.10 (dd, *J* = 15.98 Hz, 1H); ^13^C NMR (150 MHz, CDCl_3_, δ = ppm): 11.08, 23.48, 23.81, 42.57, 43.43, 93.72, 129.19, and 174.74).

### 2.5. Synthesis of Poly[(3-ethyl-1-vinyl-2-pyrrolidone)-co-(N-vinyl-2-pyrrolidone)] by RAFT Copolymerization (i.e., Copolymers C1–C6)

An example of copolymer C1 (shown in Scheme 1): the ratio of reagents was (C_2_NVP + NVP)/MHEX/AIBN = (20 + 10)/1/0.1 in EtOH ([MHEX]_0_ = 75.5 mM). C_2_NVP, NVP, MHEX, AIBN, and EtOH were added to a 25 mL Schlenk flask. The mixture was deoxygenated by three freeze–pump–thaw cycles. The flask was back-filled with nitrogen, an initial sample was taken via a syringe, and the flask was then kept at 70 °C to carry out the RAFT copolymerization. After 24 h, the reaction was stopped by placing the flask in an ice bath and exposing the contents to the air. The EtOH was removed under vacuum, and the residue was dissolved in DCM. The mixture was precipitated in n-hexane, and a white solid was obtained with a moderate yield (47.5%). The other copolymers (i.e., C2–C6) have the same procedures and were all characterized by FT-IR (see Figure S2 in the Supporting Information). The detailed information was summarized in Scheme 1 and Table 1.

### 2.6. Chain Extension of P(C_2_NVP-co-NVP)-OH with CL by ROP (i.e., BC1)

A Schlenk flask contained a heterogeneous mixture of macroinitiator C1 (*M*_n_ = 8350, *M*_w_*/M*_n_ = 1.34; 0.01 mmol), organocatalyst DPP (0.01 mmol), and distilled monomer ε-caprolactone (1 mmol). The mixture was placed in a thermostatted oil bath at 60 °C under a nitrogen atmosphere, and became homogeneous in 5 min. After a desired period, the polymerization was quenched by exposure to the air and diluted by DCM. The mixture was precipitated in n-hexane. A yellowish white solid was obtained (i.e., BC1: *M*_n_ = 10450, *M*_w_*/M*_n_ = 1.59), and dried under reduced pressure (yield 50%).

### 2.7. Characterization

^1^H NMR measurements were performed using a Varian Inova 400 NMR (Varian, Billerica, MA, USA) in CDCl_3_, and the chemical shift was calibrated by setting the internal standard of CDCl_3_ as 7.26 ppm. FT-IR spectra were measured by a Nicolet Avatar 320 FT-IR Spectrometer (Nicolet, Madsion, WI, USA); 32 scans at a resolution of 1 cm^−1^ were conducted using a KBr disk. The dissolved samples were cast onto a KBr disk and dried under vacuum. The sample chamber was purged with nitrogen to maintain the film’s dryness. The conversion of the monomers was monitored using a Hewlett Packard 5890 series II gas chromatograph (Hewlett Packard, Palo Alto, CA, USA) (GC) equipped with an FID detector, and employing a CNW CD-5 column (30 m) using anisole as an internal standard. Gel permeation chromatography (GPC) was conducted at 40 °C in dimethyl acetamide (DMAc) (flow rate: 1 mL/min), equipped with a Waters 515 pump, a Waters 410 differential refractometer, a Waters 486 absorbance detector, and two PSS SDV columns (Linear S and 100 Å pore size) to obtain *M*_n_, *M*_w_ and *M*_w_/*M*_n_. Monodisperse polystyrene (PSt) standards were used for the calibrations. The cloud point temperatures (CPs) of the copolymers were determined by transmittance measurements in a quartz cell charged with a polymer concentration of 1 mg/mL in a PerkinElmer Lambda 25 UV-vis spectrometer (PerkinElmer, Waltham, MA, USA). The transmittance was monitored at 450–700 nm, and the data were collected after the solution was equilibrated for 2 min at each temperature. The CP was defined as the temperature when the transmittance decreased to 50%. Various concentrations of block copolymer aqueous solutions were mixed with the same volume of pyrene aqueous solution with a concentration of 8 × 10^−7^ M. The mixtures were filtered through a 0.22 μm Millipore filter into a dust-free vial beforehand, to detect the critical micelle concentration (CMC). The CMC measurements were performed using a HITACHI F-2500 fluorescence spectroscopy (HITACHI, Tokyo, Japan) scanned during 300–500 nm, which conducted 250 nm as the excitation wavelength. Particle sizes were measured by laser light scattering (LLS) of a commercial spectrometer (Brookhaven Inc., Holtsville, NY, USA) equipped with a BI-200SM goniometer and a BI-9000AT digital autocorrelator. A 15 mW vertically polarized solid-state laser (630 nm) was used as the light source. At each heating/cooling step, the solutions were equilibrated for 5 min in a thermostatted bath. For the dynamic light scattering measurements, the intensity–intensity time autocorrelation function was measured in the self-beating mode. The Laplace inversion program—CONTIN—was applied, to obtain the average line width Γ with a varying scattering angle q. The apparent hydrodynamic radius (*R*_h_) was obtained by extrapolating Γ/q2 to an angle of zero, and was estimated from the Stokes-Einstein equation.

## 3. Results and Discussion

**Figure 1:**
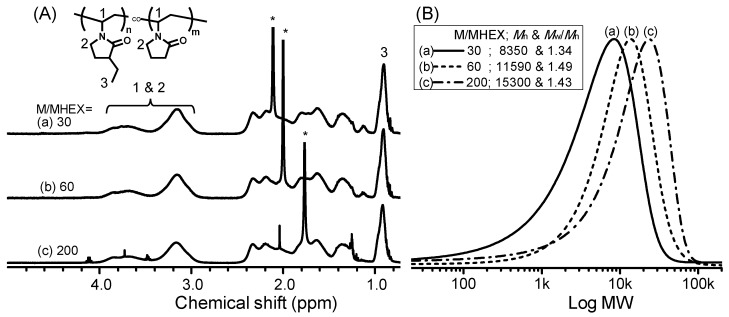
C1–C3 copolymers of (**A**) ^1^H NMR spectra (400 MHz, CDCl_3_); (**B**) GPC traces through RAFT copolymerization of *N*-vinylpyrrolidone (NVP)and 3-ethyl-1-vinyl-2-pyrrolidone (C_2_NVP) with different M/MHEX ratios (*: impurities).

**Figure 2:**
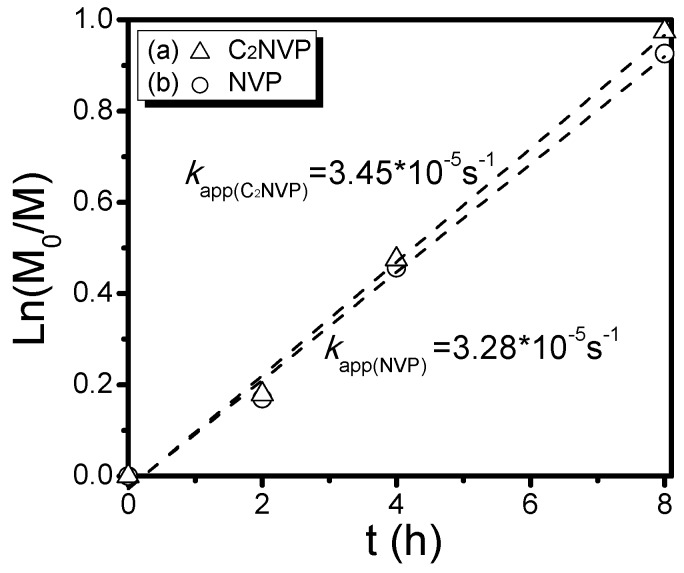
Kinetic of RAFT copolymerization of C_2_NVP and NVP (Monomers/MHEX = 200/1 in EtOH at 70 °C; [C_2_NVP]_0_ = 1.50 M and [NVP]_0_ = 0.75 M).

To synthesize copolymers with a different molecular weight (MW), we first used a molar feed of C_2_NVP/NVP = 66:34 with the different monomers/RAFT agent ratios (i.e., M/MHEX) of 30, 60, and 200 to synthesize the various MWs of the PNVP-based copolymers. In this copolymerization ratio, we expect the cloud points (CPs) of the copolymers to be close to our body temperature. The composition of the copolymers was estimated by ^1^H NMR analysis (Figure 1A) and the results are summarized in Table 1. From the ratio of the assigned 1–3 peaks (i.e., A_3_/(A_1_ + A_2_)) [51], we acquired all of the copolymers with similar content (i.e., C_2_NVP (mol %) in C1 (64.0%), C2 (64.6%) and C3 (63.9%)). Figure 1B showed the GPC traces of the related copolymers. Controlled MWs with mono-disperse and narrow molecular weight distributions (MWDs (i.e., *M*_w_/*M*_n_)] were obtained (C1: *M*_n_ = 8350, C2: *M*_n_ =11,590 and C3: *M*_n_ = 15,300; all *M*_w_/*M*_n_ < 1.5). We demonstrated one of the RAFT copolymerizations (C_2_NVP/NVP = 66:34 and M/MHEX = 200) by GC to observe the kinetic behavior. As shown in Figure 2, linear pseudo-first order relationships and moderate polymerization rates were observed from each monomer. The apparent reaction rate of C_2_NVP and NVP were *k*_app(C_2___NVP)_ = 3.45 × 10^−5^ s^−1^ and *k*_app(NVP)_ = 3.28 × 10^−5^ s^−1^, respectively. These results implied an ease of MW control and similar monomer reactivity through RAFT copolymerizations using the MHEX chain transfer agent.

**Figure 3:**
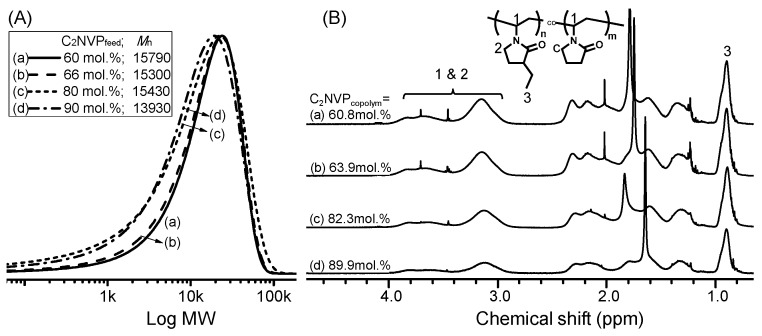
Kinetic C3–C6 copolymers of (**A**) GPC traces and (**B**) ^1^H NMR spectra (400 MHz, CDCl_3_) through RAFT copolymerization different monomer feed ratios (C_2_NVP_feed_ = 60, 66, 80, and 90 mol %; M/MHEX/AIBN = 200/1/0.1 in EtOH at 70 °C).

**Figure 4:**
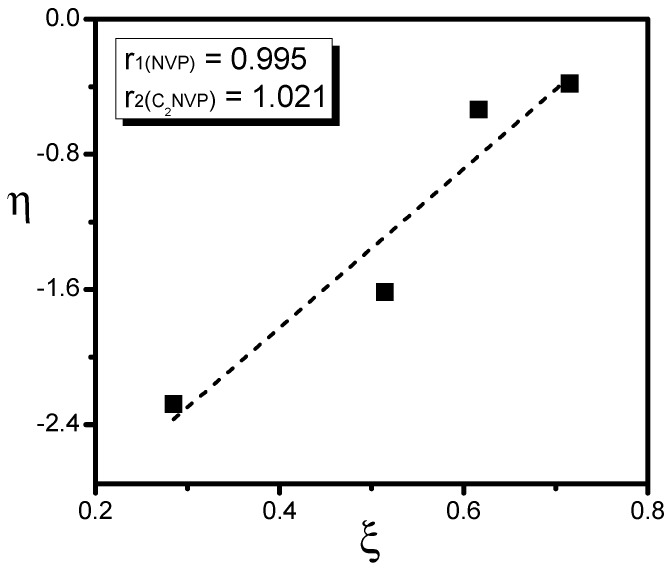
Kelen–Tüdos plot of RAFT copolymerization of C_2_NVP and NVP.

To synthesize various composition of PNVP-based copolymers, we then used a molar ratio of M/MHEX = 200 with different concentrations of C_2_NVP in feed (i.e., 60, 66, 80, and 90 mol %). As shown in Figure 3A, similar MWs of copolymers (*M*_n_ ≈ 15,000) with narrow MWDs were observed from GPC traces. Figure 3B displays the ^1^H NMR spectra of the corresponding copolymers. As mentioned above, we acquired the compositions of the copolymers based on the assigned 1–3 peaks [i.e., A_3_/(A_1_ + A_2_)] that afforded C_2_NVP_copolym_ (mol %) of C4 (60.8%), C5 (82.3%), and C6 (89.9%). To study the sequence distribution of each monomer, the reactivity ratios of C_2_NVP (*r*_C2NVP_) and NVP (*r*_NVP_) were estimated by employing the Kelen–Tüdos method. We can estimate the reactivity ratio from the well-known copolymerization equation [i.e., η = (*r*_1_ + $\frac{r_{2}}{\alpha })$ξ − $\frac{r_{2}}{\alpha }$], which contains two parameters, η and ξ, from which the reactivity ratio is derived, as described in previous studies [28,52]. Figure 4 showed the Kelen–Tüdos plot of C_2_NVP and NVP copolymerizations. Applying a linear regression of η and ξ parameters, the reactivity ratios of NVP (*r*_NVP_ = 0.995) and C_2_NVP (*r*_C_2___NVP_ = 1.021) were obtained. NVP showed a reactivity similar to C_2_NVP during RAFT copolymerization, implying that the system performed a predominantly ideal random distribution of the monomer units. The glassy transition temperatures (*T*_g_) of these copolymers were determined by DSC. As shown in Figure S3 (see Supporting Information), the *T*_g_s of C1–C3 copolymers increased from 95 to 121 °C with an increase in MW from ca. 8000 to 15,000 owing to the enhancement of chain entanglements. With different composition, the *T*_g_s of C3–C6 copolymers decreased from 130 to 114 °C with the increase in the C_2_NVP unit, which is ascribed to the lower *T*_g_ part of PC_2_NVP in copolymers (i.e., *T*_g,PC_2___NVP_ = 88 °C, shown in Figure S3). A thermogravimetric analyzer (TGA) was utilized to trace thermal stabilities of the C1–C6 random copolymers. As shown in Figure S4 (see Supporting Information), the copolymers had similar decomposition profiles, possessing over 350 °C of the 5% decomposition temperature, implying good thermal stabilities of the P(C_2_NVP-*co*-NVP) copolymers.

**Figure 5:**
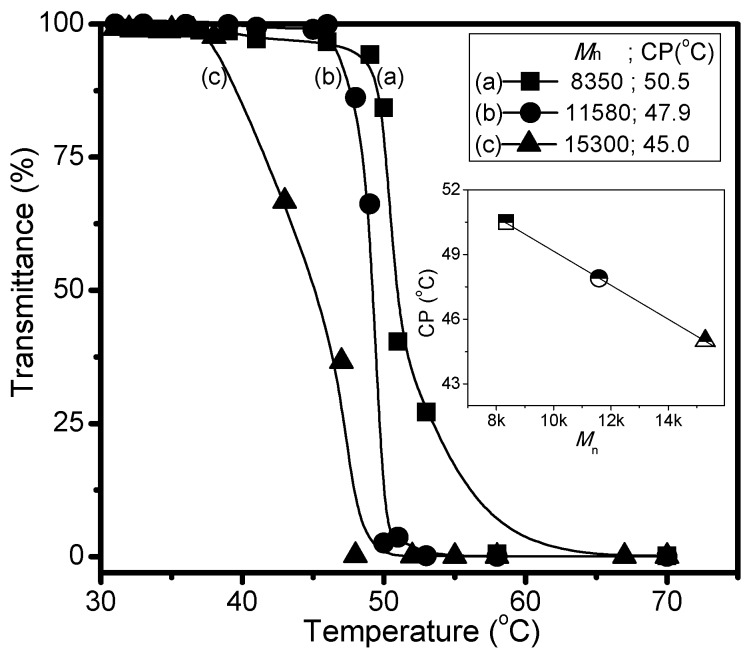
Plot of lower critical solution temperature (LCST) trends of copolymers with different molecular weights (MWs) and linear correlation curve (C_2_NVP mol % in copolymer ≈ 64%; conc. = 1 mg/mL).

**Figure 6:**
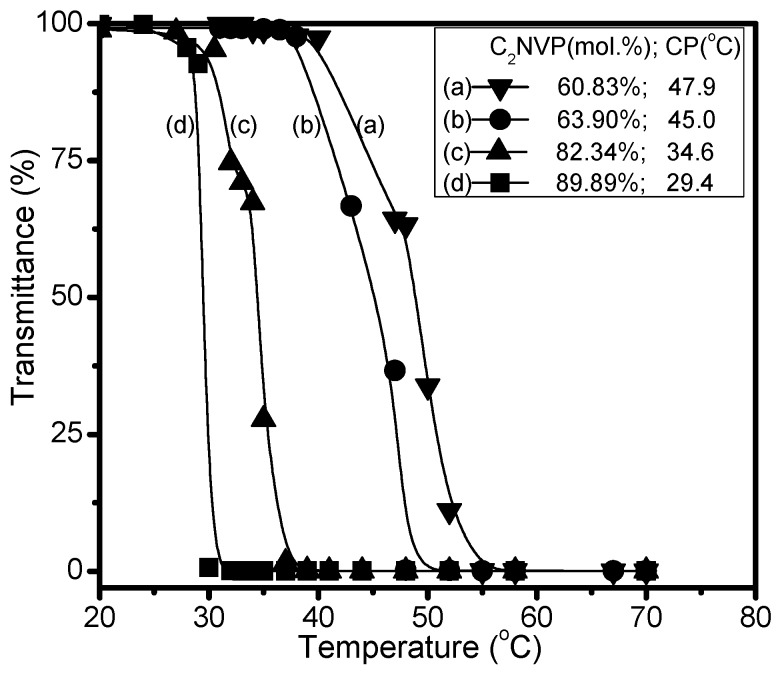
Plot of LCST trends of copolymers with different compositions (*M*_n_ ≈ 15300; conc. = 1 mg/mL).

**Figure 7:**
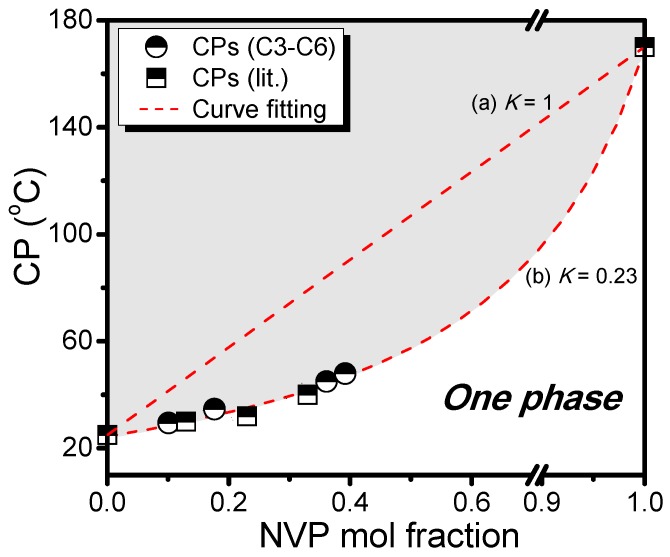
Experimental and theoretical plots of cloud points (CPs) vs. NVP molar fractions in the P(C_2_NVP-*co*-NVP) copolymers.

With control of copolymer MW and composition, the temperature-responsive properties of these random copolymers were then examined by UV/vis spectroscopy at different temperatures. LCST behaviors of the copolymer solutions (1 mg/mL) were recorded in a wavelength range of 450–700 nm. Figure S5 displays the transmittance traces with different wavelength of a representative copolymer (i.e., C3) and shows that transmittance decreased with the increase in temperature. Among the curves in Figure S5, the line of the wavelength of 500 nm (bold-line) performed a clear LCST trend (i.e., cloud point (CP) ≈ 45 °C) and was chosen to monitor the LCST trends of the copolymers C1–C6 efficiently. We then compared the MW effect on the CP of the PNVP-based random copolymers. Figure 5 showed the LCST behaviors of the copolymers with various MWs [i.e., C1 (*M*_n_ = 8350), C2 (*M*_n_ = 11,580), and C3 (*M*_n_ = 15,790)]. The CPs decreased from 50.5 to 45.0 °C with the increase in the MW of copolymers with similar composition. The CPs linearly decreased with the increase in *M*_n_ (the inset in Figure 5). It has been reported that the neat PC_2_NVP with different MWs from 2500 to 16,000 has nearly no effect on the CPs [17]. With the copolymerization of C_2_NVP and NVP, we revealed a new analogue of tunable thermosensitivity of P(C_2_NVP-*co*-NVP) copolymers through the control of the MW from ca. 8000 to 16,000. As shown in Figure 6, on the other hand, we examined the composition effect on the CPs of the copolymers with similar MWs [i.e., C3–C6 (*M*_n_ ≈ 15,000)]. The CPs of the copolymers decreased from ca. 48 to 29 °C with the increase in C_2_NVP content (i.e., from 60.8 to 89.9 mol %). The variation of CPs can be fitted by the theoretical equation [53]:

$$CP\;=\;\frac{\mu_{1}CP_{1}\;+\;K\mu_{2}CP_{2}}{\mu_{1}\;+\;K\mu_{2}}$$

where *μ*_i_ is the molar fraction of *i* monomer, *μ*_1_ + *μ*_2_ = 1, and *K* is a weighing parameter that indicates the interaction character between polymer chain and solvent molecules. Herein, the CP_2_ (i.e., CP of PNVP) cannot be determined under ordinary conditions due to the boiling point of water. The CP_2_ of 170 °C was then obtained from the previous literature [54]. Further combining CPs of our values and previous literature [19], Figure 7 shows the plot of CPs vs. NVP molar fractions with theoretical fitting lines. In the ideal case [i.e., *K* = 1 (Figure 7a line)], the trend of CPs and NVP concentrations shows a linear relationship. By fitting the experimental data (i.e., squares and circles in Figure 7), *K* = 0.23 was obtained (i.e., the red-dash curve in Figure 7b). The low value of weighing parameter derived from the P(C_2_NVP-*co*-NVP) copolymers illustrated that the introduction of the hydrophobic unit of the C_2_NVP monomer hindered the hydrophilic interaction between the polymer chain and the water and resulted in a decrease in the LCST of the copolymers.

**Figure 8:**
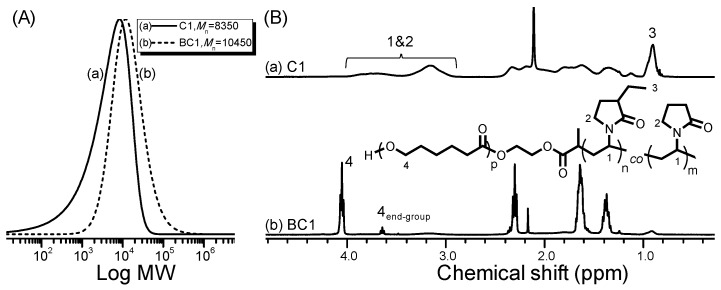
(**A**) GPC traces and (**B**) ^1^H NMR spectra (400 MHz, CDCl_3_) of (a) C1 and (b) BC1 copolymers.

We then proceeded with the chain extension of a macroinitiator possessing hydroxyl chain end through ROP of CL monomer using an organocatalyst and analyzed the results by GPC and ^1^H NMR spectroscopy. In the GPC traces (i.e., Figure 8A), the MW from the macroinitiator [i.e., (a) C1: *M*_n,GPC_ = 8350, *M*_w_*/M*_n_ = 1.34] shifted to a higher MW [i.e., (b) *M*_n,GPC_ = 10450] and remained mono-disperse with a MWD of 1.59 after chain extension. In the ^1^H NMR spectra (i.e., Figure 8B), the major peaks of C1 and BC1 could be assigned from their P(C_2_NVP-*co*-NVP) and PCL segments (i.e., Peaks 1–3 for P(C_2_NVP-*co*-NVP) and Peak 4 for PCL, respectively). We then acquired the *M*_n,NMR_ of BC1 with a value of ca. 14730. The difference in *M*_n_s measured by GPC and NMR were reasonably ascribed to the different hydrodynamic volumes between the block copolymers and the PSt calibration standards. Characterization of the BC1 data is summarized in Table S1 (see Supporting Information).

**Figure 9:**
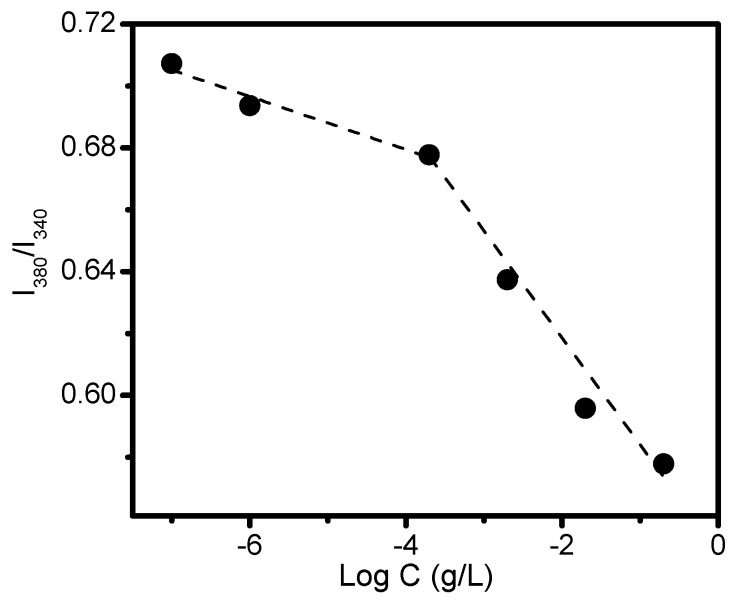
Critical micelle concentration (CMC) examination of P(C_2_NVP-*co*-NVP)-*b*-PCL block copolymer by using pyrene as a probe.

**Figure 10:**
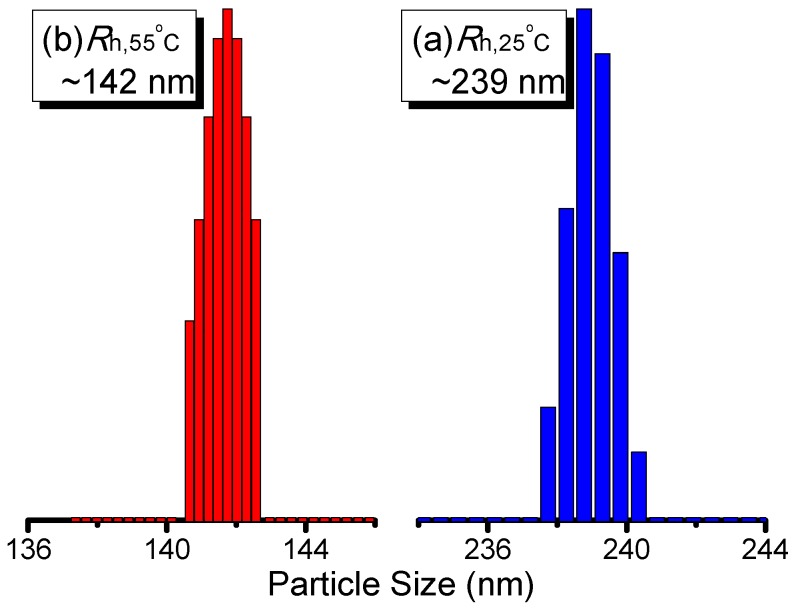
Hydrodynamic radii (*R*_h_) of P(C_2_NVP-*co*-NVP)-*b*-PCL block copolymer measured by DLS at (**a**) 25 °C and (**b**) 55 °C (conc. of BC1 = 0.002 g/L).

We thus expect that the P(C_2_NVP-*co*-NVP)-*b*-PCL block copolymer composing hydrophobic and hydrophilic segments can be applied to the fabrication of thermo-responsive micelles. To determine the critical micelle concentration (CMC) of BC1, fluorescence measurements of micelle solutions with different concentrations using pyrene as a probe were conducted. Based on the intensity ratios of 340 and 380 nm from pyrene probe and BC1 concentrations, the plot of *I*_380_/*I*_340_ vs. Log C can be obtained (as shown in Figure 9). We can acquire an obvious decline point of *I*_380_/*I*_340_ around 1.46 × 10^−4^ g/L. The rapid decrease in the intensity ratio was ascribed to the isolation of pyrene probe efficiently encapsulated through the formed micelles (i.e., CMC of BC1). To measure the thermosensitivity of the micelle size, a solution with a BC1 concentration of 0.002 g/L was monitored by dynamic light scattering (DLS) at 25 and 55 °C, respectively. At 25 °C (Figure 10a), a mono-disperse distribution with a particle size of ca. 240 nm was obtained. At 55 °C (Figure 10b), a mono-disperse particle size but with a smaller particle size of ca. 140 nm was obtained. This is a typical thermoresponsive behavior of a micelle containing an LCST segment. Namely, the hydrogen bonding of the polymer chain and water molecules breaks when the solution temperature over the LCST is elevated, and leads to shrinkages of the thermoresponsive polymer chains on the micelle surface. We thus investigated a novel analogue of the LCST block copolymer comprising a thermoresponsive P(C_2_NVP-*co*-NVP) segment (i.e., hydrophilic compartment) and a biodegradable PCL segment (i.e., hydrophobic compartment).

## 4. Conclusions 

Through the RAFT copolymerization, we first synthesized a series of well-defined P(C_2_NVP-*co*-NVP) copolymers possessing different MWs (*M*_n_ ≈ 8000–15,000) and narrow MWDs (*M*_w_/*M*_n_ < 1.5) using a difunctional chain transfer agent (i.e., MHEX). From the kinetic study, similar apparent rate constants of NVP and C_2_NVP monomers were obtained (*k*_app_ ≈ 3 × 10^−5^ s^−1^). With the copolymerization of different monomer ratios in feeds, reactivity ratios of NVP (*r*_NVP_ = 0.995) and C_2_NVP (*r*_C_2___NVP_ = 1.021) can be further estimated by the Kelen–Tüdos method, indicating predominantly ideal random copolymerization of the monomers. *T*_g_s increased with the increase in copolymer MWs and NVP contains. Regardless of MW and copolymer composition, the copolymers possessed a similar 5% decomposition temperature of ca. 350 °C. Monitoring by UV/Vis spectroscopy, the CPs decreased linearly from 50.5 to 45.0 °C with the increase in MWs from *M*_n_ = 8350 to 15,790. We revealed an analogue of tunable thermosensitivity of P(C_2_NVP-*co*-NVP) copolymers through the control of the MW. Further examining the composition effects with a similar MW, the CPs of the copolymers decreased from ca. 48 to 29 °C with the increase in C_2_NVP content (i.e., from 60.8 to 89.9 mol %). Fitting the CPs by the theoretical equation, the low value of weighing parameter (*K* = 0.23) illustrated the introduction of more hydrophobic unit of C_2_NVP monomer suppressed the hydrophilic interaction between polymer chain and water. We then successfully proceeded the chain extension by ROP of CL to the synthesis of P(C_2_NVP-*co*-NVP)-*b*-PCL amphiphilic block copolymer (*M*_n,NMR_ = 14,730 and *M*_w_/*M*_n_ = 1.59). Probing by fluorescent chromophore, CMC of the block copolymer had a value of ca. 1.46 × 10^−4^ g/L. The micelle sizes with mono-disperse distribution were ca. 240 nm at 25 °C and ca. 140 nm at 55 °C, respectively. The thermosensitive nano-carrier composing P(C_2_NVP-*co*-NVP)-*b*-PCL has a potential for use with biomaterials, drug and gene delivery systems, diagnostic imaging, and tissue engineering.

## Figures and Tables

**Table 1:** Characterization of P(C_2_NVP-*co*-NVP) copolymers (C1–C6).

No.	[M]/[I] ^a^	C_2_NVP_feed_ (mol %)	C_2_NVP_copolym_^b^ (mol %)	*M*_n_^c^	*M*_w_*/M*_n_^c^	CP (°C)
C1	30	66.7	64.0	8350	1.34	50.5
C2	60	66.7	64.6	11,580	1.42	47.9
C3	200	66.7	63.9	15,300	1.49	45.0
C4	200	60.0	60.8	15,790	1.49	47.9
C5	200	80.0	82.3	15,430	1.48	34.6
C6	200	90.0	89.9	13,930	1.43	29.4

^a^ [M]/[I] = ([C_2_NVP] + [NVP])/[MHEX]; ^b^ Estimated from peak areas in ^1^H NMR spectra (i.e., Figure 1 and Figure 3): A_3_/(A_1_ + A_2_); ^c^ *M*_n_ and *M*_w_/*M*_n_ were estimated by GPC (eluent: DMAc) using polystyrene as the standard.

## Supplementary Materials

The following are available online at www.mdpi.com/9/6/231/s1. Table S1: Characterization of P(C_2_NVP-*co*-NVP)-*b*-PCL copolymers (BC1); Scheme S1: Synthetic routes of (**a**) MHEX and (**b**) C_2_NVP; Figure S1: ^1^H NMR spectra (400 MHz, CDCl_3_) of (**A**) MHEX and (**B**) C_2_NVP compounds; Figure S2: FT-IR spectra of C1–C6 copolymers; Figure S3: DSC traces of C1–C6 copolymers (record of 2nd heating run with ramp 20 °C/min under N_2_); Figure S4: TGA traces of C1–C6 copolymers (ramp 20 °C/min under N_2_); Figure S5: LCST behaviors scanned with different wavelength (C3 copolymer: 1 mg/mL).
