# Supplementary material for: Synthesis of PNVP-Based Copolymers with Tunable Thermosensitivity by Sequential Reversible Addition–Fragmentation Chain Transfer Copolymerization and Ring-Opening Polymerization
Source: Polymers (Basel). 2017 Jun 18;9(6):231. doi: 10.3390/polym9060231 (PMC6431924; doi:10.3390/polym9060231)
Supplement: Supplementary file 1 [file polymers-09-00231-s001.pdf]

Supporting Information for

Synthesis of PNVP-based Copolymers with Tunable Thermosensitivity by Sequential Reversible Addition-Fragmentation Chain Transfer Copolymerization and Ring-Opening Polymerization

Yi-Shen Huang, Jem-Kun Chen, Tao Chen and Chih-Feng Huang*

Table S1. Characterization of P(C_2NVP-*co*-NVP)-*b*-PCL copolymers (BC1).

No.	[CL]/[C1][a]	$M_{n,GPC}$[b]	M_w/M_n[b]	$M_{n,NMR}$	CMC[c] (10^{-4} g/L)	$R_{h,25\,°C}$[d] (nm)	$R_{h,55\,°C}$[d] (nm)
**BC1**	100	10450	1.59	14730	1.46	239	142

[a] C1: M_n = 8350 and M_w/M_n = 1.34.

[b] M_n and M_w/M_n were estimated by GPC (eluent: DMAc) using polystyrene as the standard.

[c] Estimated by fluorescence spectra (λ_{ex} = 250 nm) in the region of 300–500 nm with different concentration of block copolymer using pyrene as a probe (4×10^{-7} M).

[d] Measured by dynamic light scattering (DLS) (conc. of BC1 = 0.002 g/L).

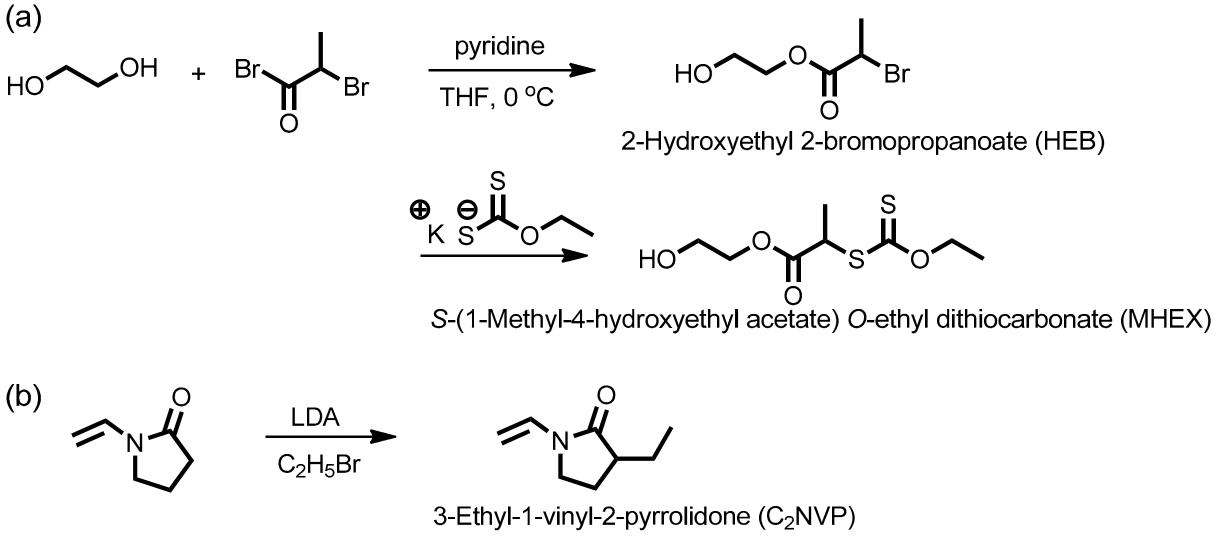

Scheme S1. Synthetic routes of (**a**) MHEX and (**b**) C_2NVP.

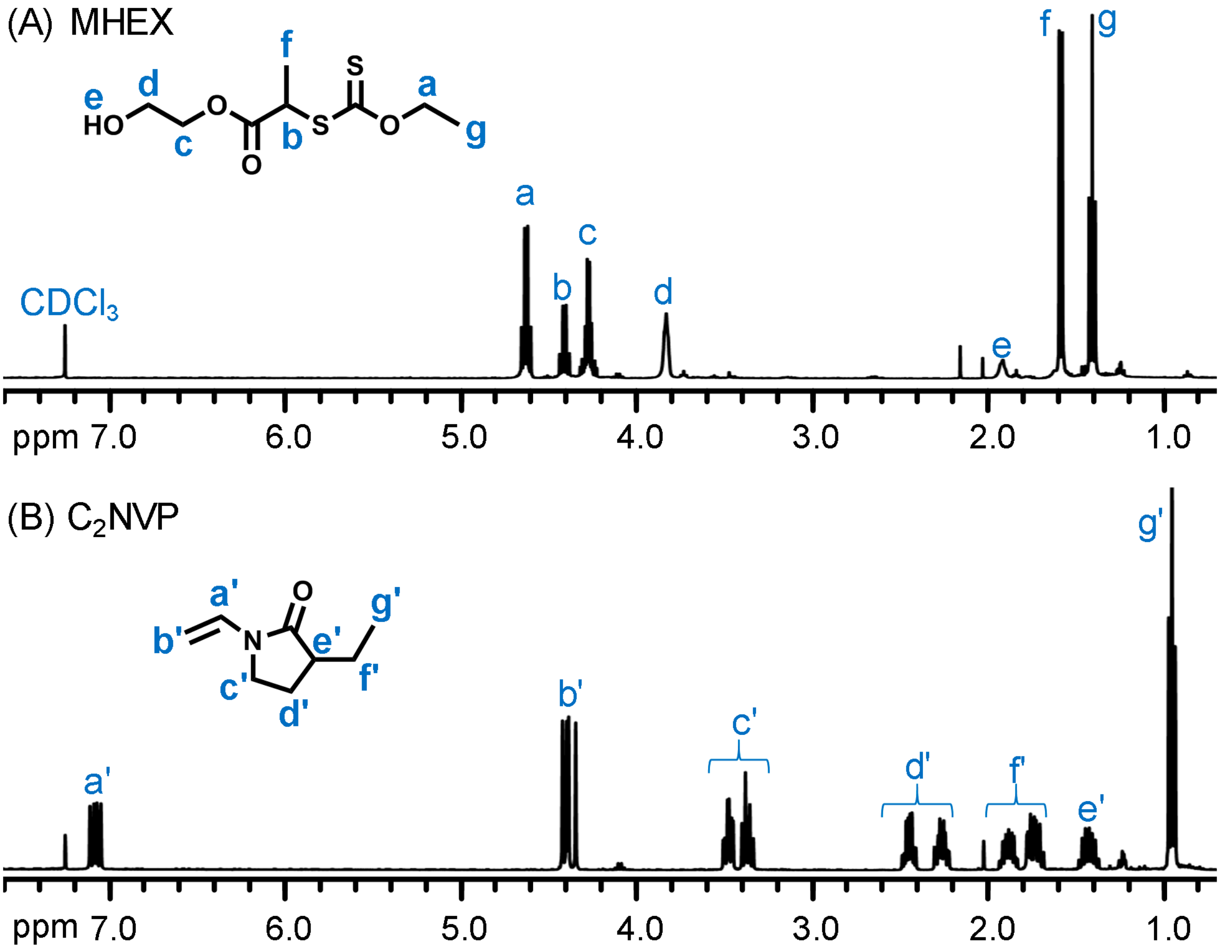

Figure S1. ^{1}H NMR spectra (400 MHz, $CDCl_3$) of (**A**) MHEX and (**B**) C_2NVP compounds.

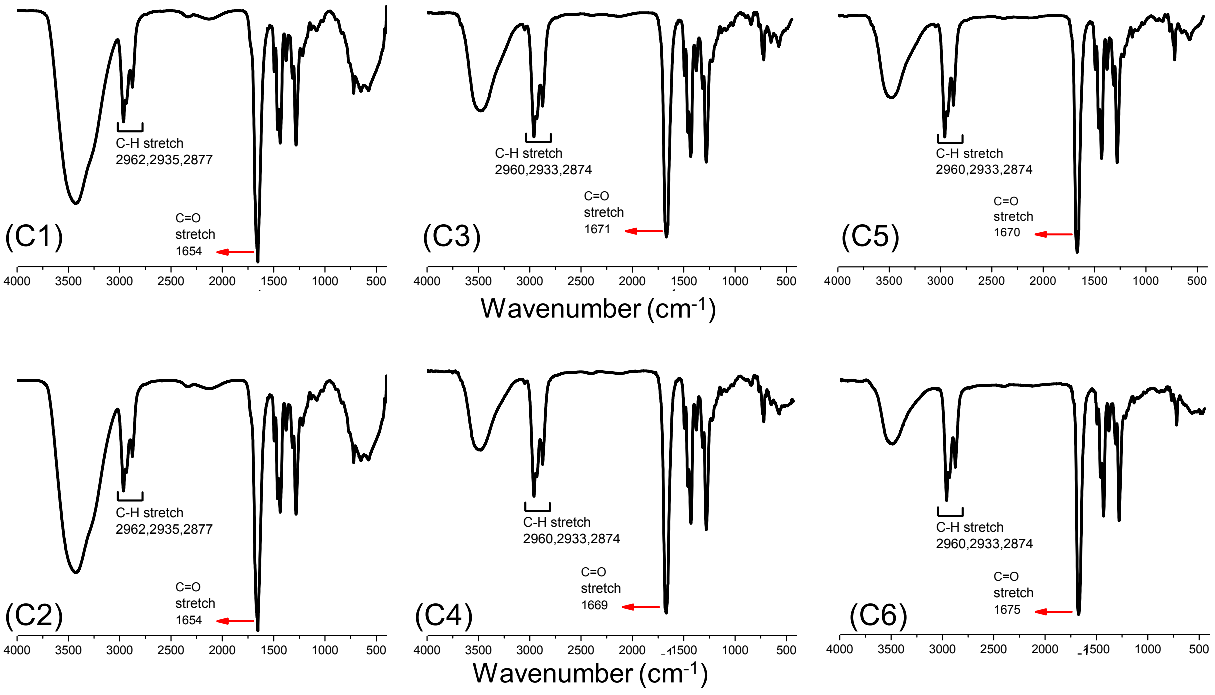

Figure S2. FT-IR spectra of C1–C6 copolymers.

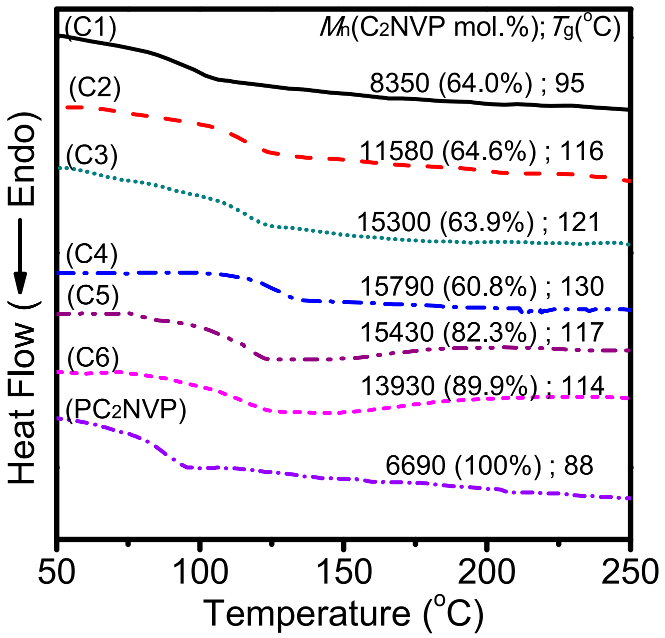

Figure S3. DSC traces of C1–C6 copolymers (record of 2nd heating run with ramp 20 °C/min under N_2).

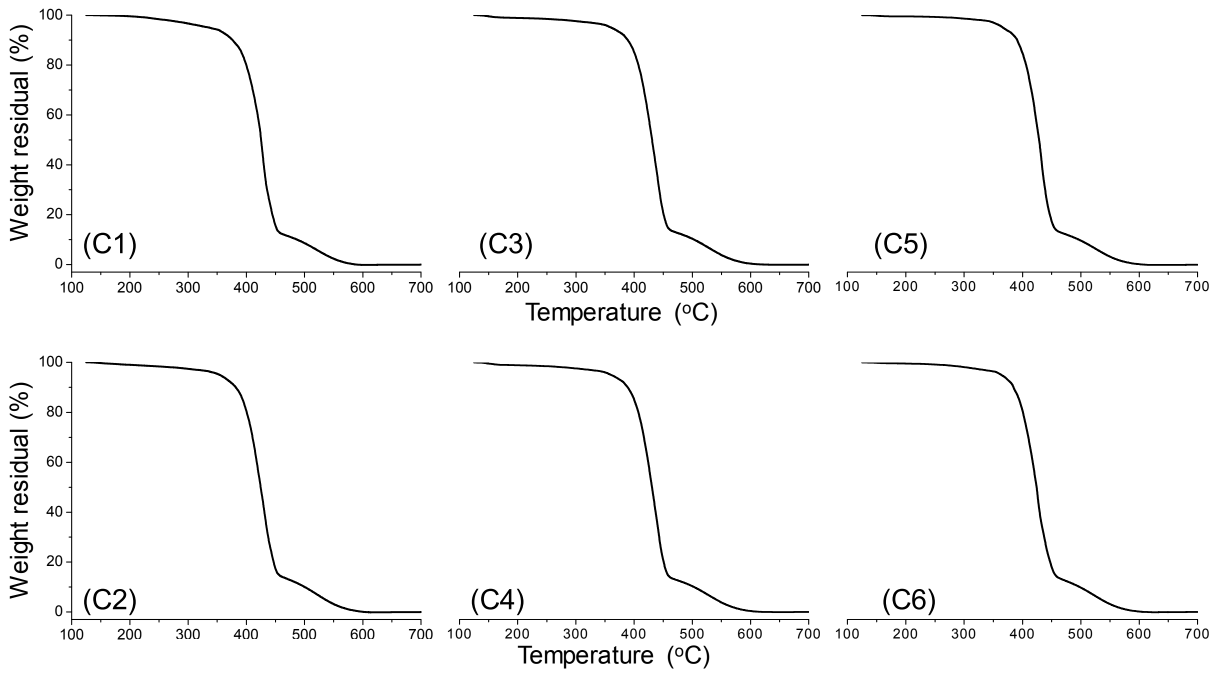

Figure S4. TGA traces of C1–C6 copolymers (ramp 20 °C/min under N_2).

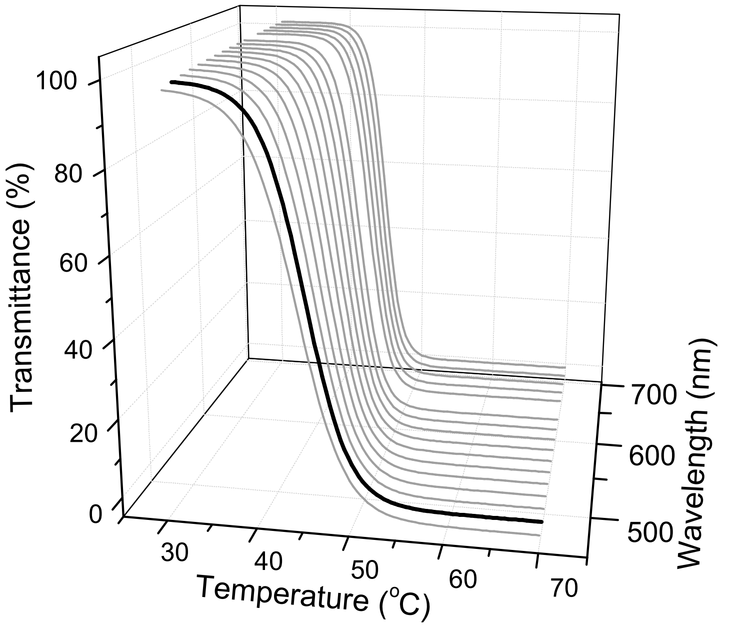

Figure S5. LCST behaviors scanned with different wavelength (C3 copolymer: 1 mg/mL).
